# A novel tool for predicting the risk of central lymph node metastasis in patients with papillary thyroid microcarcinoma: a retrospective cohort study

**DOI:** 10.1186/s12885-022-09655-5

**Published:** 2022-06-02

**Authors:** Qian-wen Luo, Shan Gao, Xiao Lv, Si-jia Li, Bo-fang Wang, Qing-qing Han, Yun-peng Wang, Quan-lin Guan, Tao Gong

**Affiliations:** 1 The First Clinical Academy of Lanzhou University, Lanzhou, 730000 Gansu China; 2 Department of Gland Surgery, HeBei General Hospital, Shijiazhuang, 050051 HeBei China; 3 Department of Cardiovascular Medicine, People’s Hospital of Dongxihu District, Wuhan, 430040 Hubei China; 4 The Second Clinical Academy of Lanzhou University, Lanzhou, 730000 Gansu China; 5 Department of surgical oncology, The Lanzhou University First Hospital, Lanzhou, 730000 Gansu China

**Keywords:** Papillary thyroid microcarcinoma, Central lymph node metastasis, Influencing factors, Nomogram, Risk

## Abstract

**Introduction:**

Central lymph node status in papillary thyroid microcarcinoma (PTMC) plays an important role in treatment decision-making clinically, however, it is not easy to predict central lymph node metastasis (CLNM). The present work focused on finding the more rational alternative for evaluating central lymph node status while identifying influencing factors to construct a model to predict CLNM incidence.

**Methods:**

In this study, we retrospectively analyzed the typical sonographic and clinicopathologic features of 546 PTMC patients who underwent surgery, among which, the data of 382 patients were recruited in the training cohort and that of 164 patients in the validation cohort. Based on the outcome of the training cohort, significant influencing factors were further identified through univariate analysis and were considered as independent variables in multivariable logistic regression analysis and incorporated in and presented with a nomogram.

**Results:**

In total, six independent predictors, including the age, sex, tumor size, multifocality, capsular invasion, Hashimotos thyroiditis were entered into the nomogram. Both internal validation and external validation revealed the favorable discrimination of our as-constructed nomogram. Calibration curves exhibited high consistency. As suggested by decision-curve analyses, the as-constructed nomogram might be applied in clinic. Besides, the model also distinguished patients according to risk stratification.

**Conclusions:**

The novel nomogram containing remarkable influencing factors for CLNM cases was established in the present work. The nomogram can assist clinicians in clinical decision-making.

## Introduction

Thyroid cancer (TC) shows an increasing incidence globally within the last decades [1, 2], primarily, papillary thyroid cancer (PTC), and it is mostly due to the advancement and popularization of neck diagnostic imaging, ultrasonography (US), as well as US-assisted fine-needle aspiration biopsy (FNAB) [1, 3]. Despite the increased incidence of papillary thyroid cancer, its mortality rate is relatively low and stable [4]. TC currently ranks fifth among the most common cancer diagnosed in women [5, 6]. Additionally, it is estimated that it will be the second leading cancer diagnosed in women and the ninth leading cancer diagnosed in men by 2030 [5, 6].

According to the classification system by the World Health Organization, papillary thyroid microcarcinoma (PTMC) refers to the PTC that is ≤10 mm. PTMC generally presents the idle clinical process and has a better prognosis than many other malignancies, with a five-year disease-specific survival of greater than 98% [3], a subset of papillary thyroid microcarcinoma (PTMC) usually shows potentially aggressive behavior [7]. Central lymph node metastasis (CLNM) has become frequently seen in PTMC cases and has been considered as the adverse factor that predict metastases and relapse [8]. Presently, lymph node dissection (LND) has become a common surgical strategy in clinical lymph node-positive (cN1) PTMC patients. Nevertheless, it remains controversial about prophylactic central neck dissection (pCND)‘s function among PTMC patients with negative clinical lymph node (cN0). The discrepancies between different guidelines have been noted.

The 2015 British Thyroid Association (BTA) and American Thyroid Association (ATA) guidelines indicate that routine prophylactic central neck dissection (pCND) is not performed on T1 and T2 or noninvasive (cN0) PTMC patients [9, 10], but patients with extrathyroidal extension or with tumors greater than 4 cm need pCND. This differs from the guidelines in China and Japan. According to the 2016 edition of the consensus and guidelines of experts on the diagnosis and treatment of papillary thyroid microcarcinoma in China, pCND is feasible in patients with cN0 PTMC [11]. According to the consensus statement from the Japan Association of Endocrine Surgery Task Force on Management for papillary thyroid microcarcinoma, active surveillance (AS) can be performed in adult patients with low-risk PTMC. However, patients with LNM, distant metastasis, and invasion of adjacent organs (especially the trachea and recurrent laryngeal nerve) should be treated surgically [12]. Moreover, the latest guidelines released by diverse international entities change their efforts on restricting the redundant selection and management for diagnosis and treatment of thyroid cancers [9, 13]. Hence, it is necessary to determine whether cN0 PTMC patients need pCND.

Currently, ultrasonography (US) has been an important approach that can assess the CLNM in PTC patients non-invasively. However, whether US is a good choice for the evaluation of CLNM remains debatable, especially for imbalanced sensitivity (23.0–61.0%) and specificity (86.8–97.0%) [14–17]. A meta-analysis revealed that US performed poorly while determining the presence of CLNM [18]. The high rate of CLNM and the low sensitivity of ultrasonography make it challenging to evaluate the factors associated with CLNM. Considering that the status of the lymph node is important for making clinical decisions, many scholars have studied the preoperative ultrasonographic features of CLNM [19, 20]. Therefore, the accurate identification of the sonographic and clinicopathologic features for predicting CLNM in patients with PTMC is the crucial first step in making a therapeutic decision and the prognosis of the patients.

There are no quantified standards in clinical practice yet that can predict the probability of CLNM, the evaluation of the risk of CLNM is only through the surgeon’s experience and general guidelines.

Hence, the aims of our study were the identification of the factors that significantly influence CLNM and the development of a nomogram for predicting CLNM among individuals.

## Materials and methods

### Selection of the patients

**Fig. 1:**
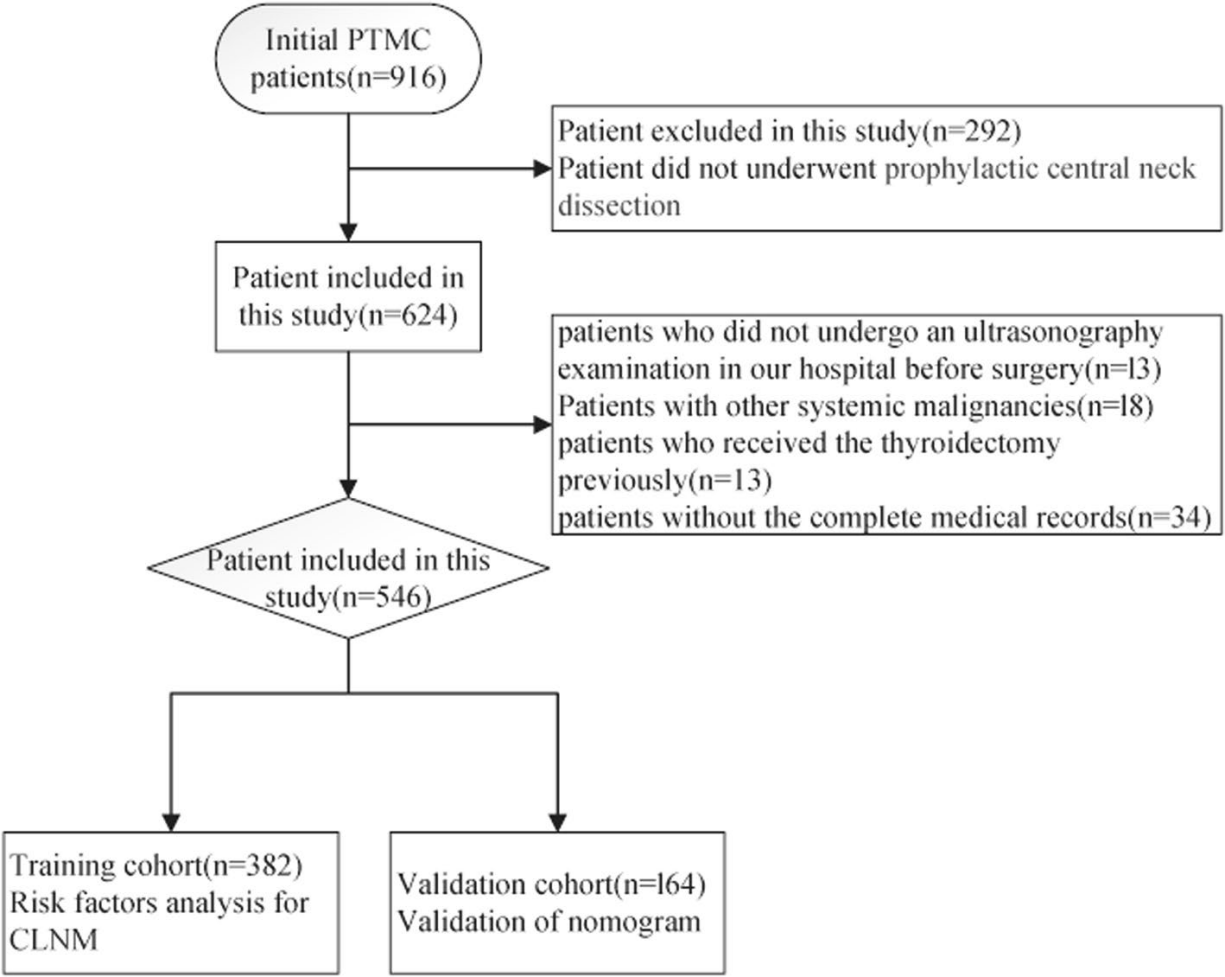
The Flow diagram of study design. PTMC:Papillary thyroid microcarcinoma; CLNM: central lymph node metastasis

We obtained materials for the present work at Hebei General Hospital, China. From 1st October 2014 to 1st October 2020, 916 papillary thyroid microcarcinoma patients underwent surgery at our hospital. Among them, we examined 546 cases receiving pCND in the present retrospective work (Fig. 1). Cases were randomized as training and validation cohorts in a 7:3 ratio.

The inclusion criteria of the participants were shown below: (1) cases undergoing the first thyroid surgery, and a postoperative pathology confirmed as PTMC; (2) patients who underwent bilateral or unilateral central lymph node dissection; (3) patients who were examined by ultrasonography and laboratory tests at our hospital before the operation; (4) patients who did not have a history of radiation and other head and neck malignant tumors.

The exclusion criteria of the participants were as follows: (1) patients who were not examined by ultrasonography at our hospital before surgery; (2) patients with other systemic malignancies; (3) patients who had received a thyroidectomy previously; (4) patients without complete medical records; (5) patients with poor cardiopulmonary function; (6) patients who had undergone levothyroxine supplementation therapy.

### Ultrasound analysis

Based on the above-mentioned patient screening standards, we selected 546 PTMC patients for the study. Ultrasonography was performed by two deputy chief physician of the ultrasound department who were professionally trained and with extensive clinical experience. In cases that the experts had different opinions, the third senior expert chief physician was involved in the discussion and a final decision was made. The parameters that were noted for further analysis were age, sex, thyroid stlmulating hormone (TSH) level before surgery, aspect ratio (the anteroposterior dimension/the transverse diameter), tumor size, capsular invasion, calcification, multifocality, Hashimoto’s thyroiditis (HT), nodular goiter, and central lymph node metastasis (CLNM).

### Selection of the predictors

To construct the model for statistical analysis, we considered the status of central lymph node metastasis as the response variable, with two categories, N0 and N1 (N1a + N1b). The parameters which we considered as factors that could influence the involvement of the central lymph node in PTMC included age, sex, preoperative TSH level, tumor size, aspect ratio (A/T), capsular invasion, calcification, multifocality, Hashimoto’s thyroiditis (HT), and nodular goiter. According to the most recent revision (eighth edition) of TNM by the American Joint Committee on Cancer (AJCC), the threshold of age for high-risk of disease-specific mortality was updated from 45 years to 55 years; patients in this study were divided into three age groups, below 45 years, between 45 and 55 years, and above 55 years. Tumor size, which indicates the maximum diameter of a tumor, was recorded as a continuous variable and then converted into a categorical variable with two categories: < 0.65 cm and ≥ 0.65 cm. Multifocality was categorized either as solitary lesions or multifocal lesions. A solitary lesion was defined as the presence of a tumor at one site within the thyroid, while multifocal lesions indicated the presence of tumors at two or more sites in the thyroid gland. The capsular invasion of the primary PTMC was classified into two groups, and the other variables were also divided into two groups.

### Statistical analysis

We took the SPSS statistical package (version 23.0, IBM Corp.) and the R software (version 3.5.2) for the data analysis. The mean ± standard deviation (SD) was used to represent continuous variables. Besides, categorical variables were shown as the number and percentages of cases, and odds ratio (OR) which had 95% confidence intervals (CI). In the current work, the Student’s t-test was performed to compare the continuous variables. Meanwhile, to investigate the categorical variables, the Chi-squared test (or Fisher’s exact test in specific scenarios) was carried out when making the comparison of the differences among a variety of groups. The risk factors in association with CLNM were analyzed based on univariate analysis and multivariable logistic regression modeling. In order to confirm the independent influential factors, the multivariable logistic regression analysis was conducted by choosing all variables possessing a *p*-value less than 0.05 in the univariate analysis. Regarding the multivariable logistic analysis, variables which are not statistically significant were eliminated from the final model. At the same time, those possessing a p-value less than 0.05 were regarded as independent risk factors and chosen in the final model. Risk stratification of the model by K-Means cluster analysis. The significance level for all statistical tests was set at 0.05. All statistical tests were two-sided.

### Nomogram establishment and verification

In order to develop a precise approach to accurately and quantitatively estimate the risk of CLNM in individuals, we established a nomogram incorporating each identified risk factor which were discovered from multivariable regression based on training cohort with R software “rms” package. We just considered those risk factors that were discovered through multivariable regression in our eventual model for nomogram construction, rather than variables with statistical significance upon univariate analysis but not upon multivariable analysis. Upon multivariate regression, we converted the risk factors’ regression coefficients into the certain values ranging from 0 to 100. We assessed the discrimination ability, which indicated nomogram prediction accuracy, through the receiver operating characteristic (AUC) curve and concordance index (C-index) for internal/external verification. We estimated the nomogram prediction performance based on training cohort, and later verified it based on validation cohort. We also conducted calibration for comparing the consistency of probability of CLNM estimated by our constructed nomogram with the actual measurements. This study then acquired calibration curves for the two cohorts and later adopted Hosmer-Lemeshow test for assessment. To evaluate the ultrasonographic nomogram’s apparent incremental utility, we evaluated the nomogram’s clinical utility by the decision-curve analyses (DCA).

### Risk stratification

We distinguished the patients based on the risk scores. In the training cohort and validation cohort, we established risk stratification based on patient risk scores, and divided patients into three subgroups: low score risk group, medium score risk group and high score risk group, and we also compared the positive rate of CLNM between different subgroups.

## Results

### Demographics and ultrasound characteristics of PTMC patients

The general characteristics of 546 patients with PTMC, including age, sex, thyroid stlmulating hormone (TSH) level before surgery, aspect ratio (the anteroposterior dimension/the transverse diameter), tumor size, capsular invasion, calcification, multifocality, Hashimoto’s thyroiditis (HT), nodular goiter, are presented in Table 1.

**Table 1:** General characteristics of 546 patients with PTMC

Item	Overall	CLNM(%)		*P*.Value
		negative (*n* = 383)	positive(*n* = 163)	
Age (years)				< 0.001***
< 45	205 (37.5%)	125 (32.6%)	80 (49.1%)	
45 ≤ age<55	194 (35.5%)	140 (36.6%)	54 (33.1%)	
≥ 55	147 (26.9%)	118 (30.8%)	29 (17.8%)	
Sex:				0.522
Female	420 (76.9%)	298 (77.8%)	122 (74.8%)	
Male	126 (23.1%)	85 (22.2%)	41 (25.2%)	
TSH	2.10 [1.28;3.12]	2.15 [1.31;3.12]	2.07 [1.23;3.11]	0.538
Tumor_size				< 0.001***
< 0.65	324 (59.3%)	260 (67.9%)	64 (39.3%)	
≥ 0.65	222 (40.7%)	123 (32.1%)	99 (60.7%)	
Aspect_ratio				0.380
< 1	264 (48.4%)	180 (47.0%)	84 (51.5%)	
≥ 1	282 (51.6%)	203 (53.0%)	79 (48.5%)	
Capsular invasion				< 0.001***
No	487 (89.2%)	354 (92.4%)	133 (81.6%)	
Yes	59 (10.8%)	29 (7.57%)	30 (18.4%)	
Calcification				0.006**
No	200 (36.6%)	155 (40.5%)	45 (27.6%)	
Yes	346 (63.4%)	228 (59.5%)	118 (72.4%)	
Multifocality				< 0.001***
Multiple	192 (35.2%)	106 (27.7%)	86 (52.8%)	
Solitary	354 (64.8%)	277 (72.3%)	77 (47.2%)	
Hashimotos thyroiditis				0.020*
No	445 (81.5%)	302 (78.9%)	143 (87.7%)	
Yes	101 (18.5%)	81 (21.1%)	20 (12.3%)	
Nodular goiter				0.757
No	322 (59.0%)	228 (59.5%)	94 (57.7%)	
Yes	224 (41.0%)	155 (40.5%)	69 (42.3%)	

*TSH* thyroid stimulating hormone, *HT* Hashimoto’s thyroiditis, *CLNM* central lymph node metastasis; *P < 0.05; **P < 0.01; ***P < 0.001

### Analysis of demographic and ultrasound characteristics

Compared to non-CLNM patients, patients in CLNM group had higher levels of preoperative TSH. There was no difference in the aspect ratio (A/T) between the two groups (48.50% vs. 53.00%). Additionally, the rate of the capsular invasion was significantly different between the two groups (18.40% vs. 7.90%). The rate of calcification in CLNM group was higher than non-CLNM groups (72.40% vs. 59.50%). The rate of multifocality was significantly different between the two groups (52.80% vs. 27.70%). Among HT patients, the patients with CLNM had a lower frequency of HT compared to the non-CLNM patients (12.30% vs. 21.10%). The rate of nodular goiter between the two groups was similar (42.30% vs. 40.50%).

### The patient features of both cohorts

Table 2 displays patient features for both cohorts. Positive CLNM rate was 29.80% in training cohort and 29.90% in validation cohort(*P* > 0.05). No significant difference in baseline clinicopathological and sonographic features was observed between both cohorts, which verified homogeneity in baseline data between two cohorts.

**Table 2:** Characteristics of patients in the Training cohort and test cohort

Item	Training cohort (*n* = 382)	Test cohort (*n* = 164)	*t/U/x*^*2*^	*P*
Age (years)			0.308	0.095
< 45	141 (36.90%)	64 (39.00%)		
45 ≤ age<55	146 (38.20%)	48 (29.30%)		
≥ 55	95 (24.90%)	52 (31.70%)		
Sex			0.720	0.557
Female	297 (77.70)	123 (75.00)		
Male	85 (22.30)	41 (25.00)		
TSH	2.09 (1.30,3.10)	2.19 (1.26,3.17)	0.930	0.926
Tumor size (cm)			0.941	0.427
≥ 0.65	160 (41.90%)	62 (37.80%)		
< 0.65	222 (58.10%)	102 (62.20%)		
Aspect ratio(A/T)			0.11	0.601
≥ 1	194 (50.80%)	88 (53.70%)		
< 1	188 (49.20%)	76 (46.30%)		
Capsular invasion			0.881	0.593
Yes	39 (10.20%)	20 (12.20%)		
No	343 (89.80%)	144 (87.80%)		
Calcification			0.165	0.489
Yes	238 (62.30%)	108 (65.90%)		
No	144 (37.70%)	56 (34.10%)		
Multifocality			0.029	1.000
Yes	134 (35.10%)	58 (35.40%)		
No	248 (64.90%)	106 (64.60%)		
HT			3.019	0.780
Yes	69 (18.10%)	32 (19.50%)		
No	313 (81.90%)	132 (80.50%)		
Nodular goiter			0.006	0.423
Yes	152 (39.80%)	72 (43.90%)		
No	230 (60.20%)	92 (56.10%)		
CLNM			0.004	1.000
Yes	114 (29.80%)	49 (29.90%)		
No	268 (70.20%)	115 (70.10%)		

*TSH* thyroid stimulating hormone, *HT* Hashimoto’s thyroiditis, *CLNM* central lymph node metastasis

### Predictors of CLNM in PTMC patients

**Fig. 2:**
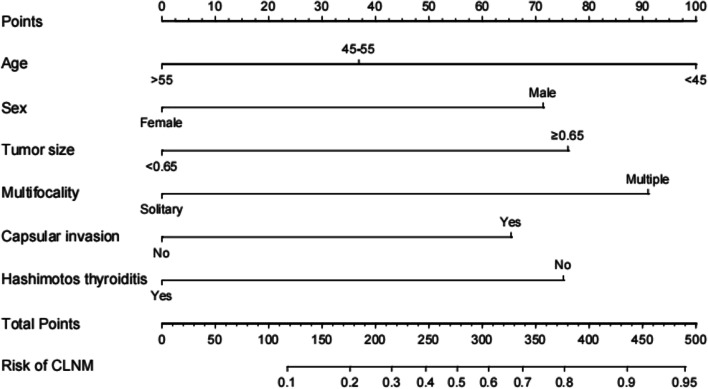
Nomogram in estimating the risk of CLNM in PTMC. To assess metastasis risk:(1) drawing a vertical line from each variable axis to the “Points” axis. (2) adding the points of each variable and locate them on the “Total Point” axis. (3) Then drawing a vertical line from the “Total Points” axis to the axis labeled “Risk” to obtain the individual probability of central lymph node metastasis. PTMC:papillary thyroid microcarcinoma;CLNM:central lymph node metastasis

Figure 2 and Table 3 list results of univariate as well as multivariate regression. Upon the univariate analysis, six significant factors were found, including age, sex, tumor size, capsular invasion, multifocality, and Hashimoto’s thyroiditis. Six risk factors (age, tumor size, capsular invasion, multifocality, and HT) were incorporated in regression analysis upon the multivariable analysis. At last, age (OR 0.418, 95% CI:0.235–0.743 / (OR 0.251,95% CI:0.125–0.506)), sex (OR 2.681, 95% CI:1.454–4.943), tumor size (OR 2.858, 95% CI: 1.728–4.727), capsular invasion (OR 2.466, 95% CI: 1.123–5.415), multifocality (OR 3.516, 95% CI: 2.095–5.901), and Hashimoto’s thyroiditis (OR 0.354, 95% CI: 0.164–0.766) were found to be the independent risk factors for CLNM.

**Table 3:** Univariate analysis and multivariate analysis in the training cohort

Item	Univariate analysis		Multivariate analysis	
	OR(95% CI)	*P* value	OR(95% CI)	*P* value
Age		0.002**		<0.001***
< 45	Reference		Reference	
45 ≤ age<55	0.519 (0.315–0.855)	0.010*	0.418 (0.235–0.743)	0.003**
≥ 55	0.368 (0.201–0.675)	0.001**	0.251 (0.125–0.506)	<0.001***
Sex		0.041*		0.002**
Female	Reference		Reference	
Male	1.692 (1.021–2.805)		2.681 (1.454–4.943)	
TSH	1.000 (0.989–1.011)	0.977	Reference	
Tumor size (cm)		<0.001***		<0.001***
< 0.65	Reference		Reference	
≥ 0.65	3.506 (2.218–5.544)		2.858 (1.728–4.727)	
Aspect ratio(A/T)		0.517		
< 1	Reference			
≥ 1	0.865 (0.558–1.341)			
Capsular invasion		0.001**		0.024*
No	Reference		Reference	
Yes	3.316 (1.60–6.148)		2.466 (1.123–5.415)	
Calcification		0.067		
No	Reference			
Yes	1.547 (0.97–2.468)			
Multifocality		<0.001***		<0.001***
No	Reference		Reference	
Yes	2.913 (1.848–4.591)		3.516 (2.095–5.901)	
HT	Reference	0.003**		0.008**
No			Reference	
Yes	0.341 (0.167–0.693)		0.354 (0.164–0.766)	
Nodular goiter		0.934		
No	Reference			
Yes	0.981 (0.627–1.536)			

*TSH* thyroid stimulating hormone, *HT* Hashimoto’s thyroiditis; *P < 0.05; **P < 0.01; ***P < 0.001

### Nomogram establishment and verification for predicting CLNM

We established the CLNM prognostic nomogram by incorporating six independent influencing factors (Fig. 2). Later, we determined points for all predicting factors by plotting the upward straight line from individual predicting factors that showed certain status to “Point” axis, in nomogram application. Thereafter, we added each point of those six  predicting factors to determine the overall points for separate patients. We later drew the downward straight line from “Total-Point” axis to “Probability-of-CLNM” axis to determine CLNM incidence among PTMC cases.

**Fig. 3:**
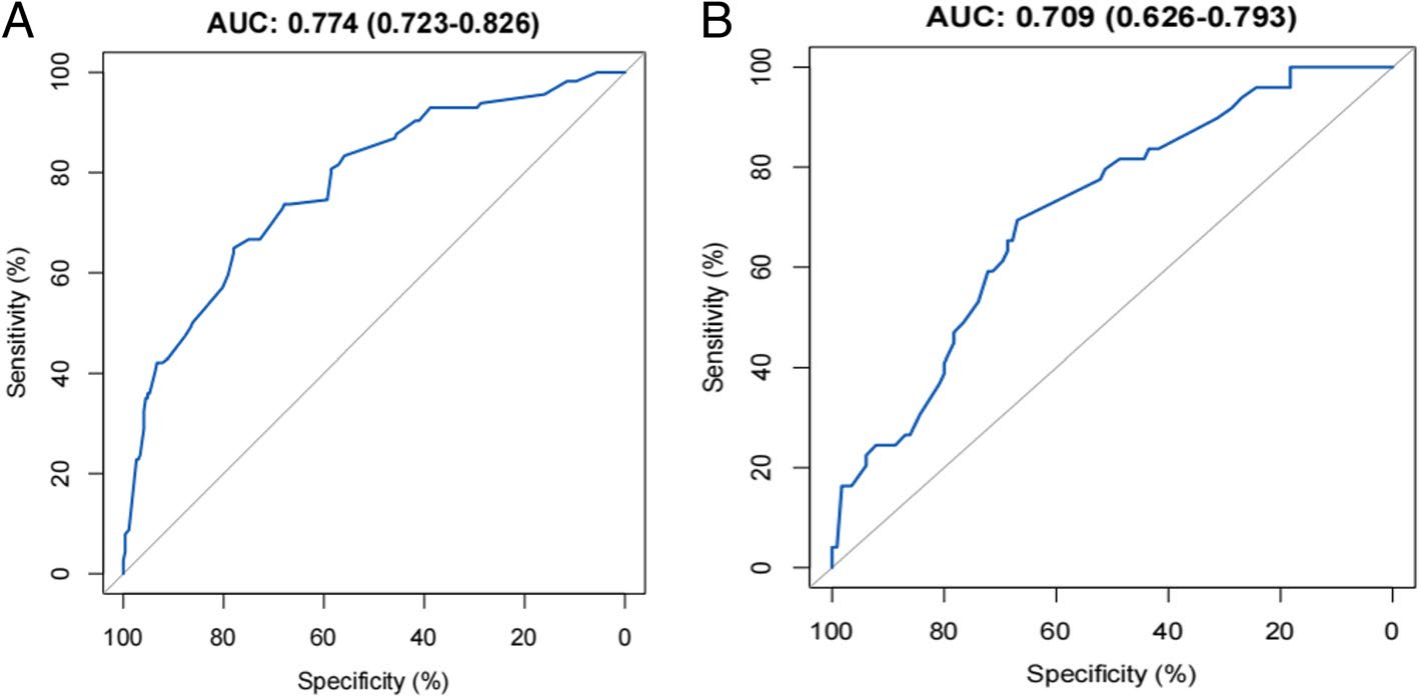
ROC curve for the predictive of preoperative ultrasonographic features. **A** The predictive model of CLNM in training cohort was accurate and discriminating, with AUC of 0.774. **B** The predictive model of CLNM in validation cohort was accurate and discriminating, with AUC of 0.709. AUC = area under ROC curve; ROC = receiver operating characteristic

**Fig. 4:**
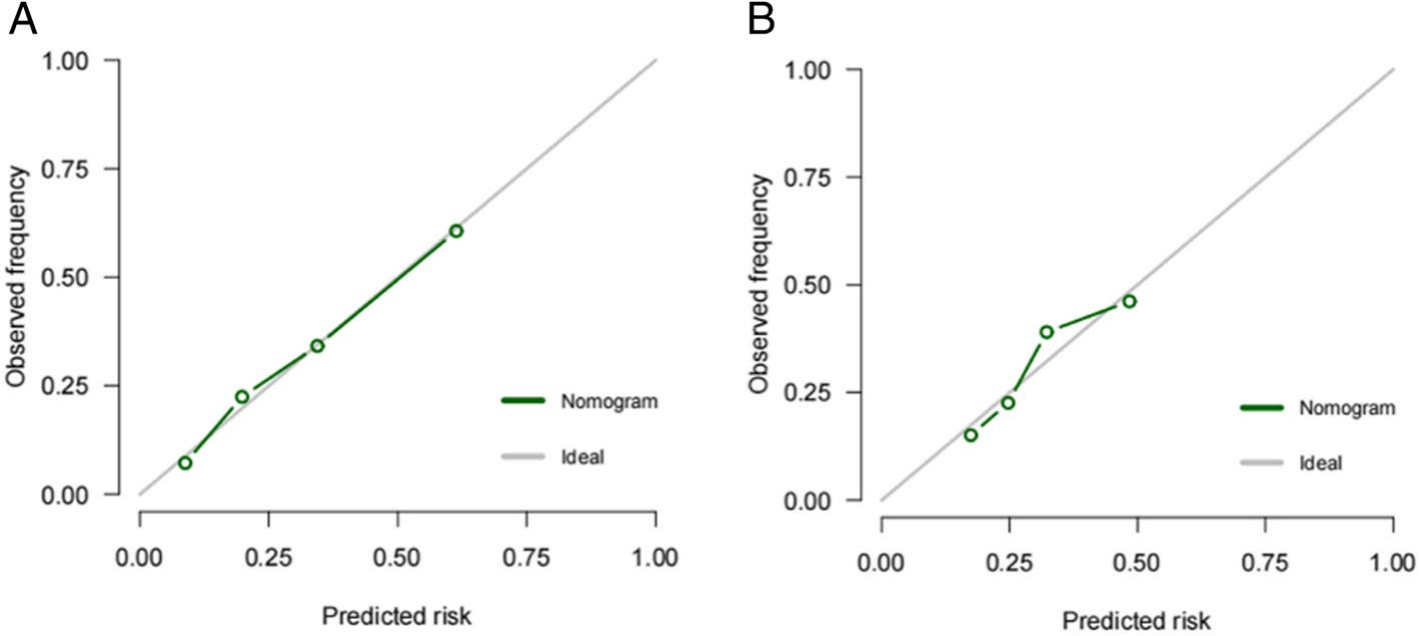
Calibration curves of the nomogram for predicting CLNM in PTMC patients. **A** Calibration curve of the nomogram for training cohort. **B** Calibration curve of the nomogram for validation cohort. The x-axis represents the predicted CLNM. The y-axis represents the actual CLNM. The diagonal dotted line stands for a perfect prediction using an ideal model. We drew the solid line to represent the performance of the nomogram, of which the closer fit to the diagonal dotted line represents the better prediction of the nomogram. PTMC:papillary thyroid microcarcinoma;CLNM:central lymph node metastasis

**Fig. 5:**
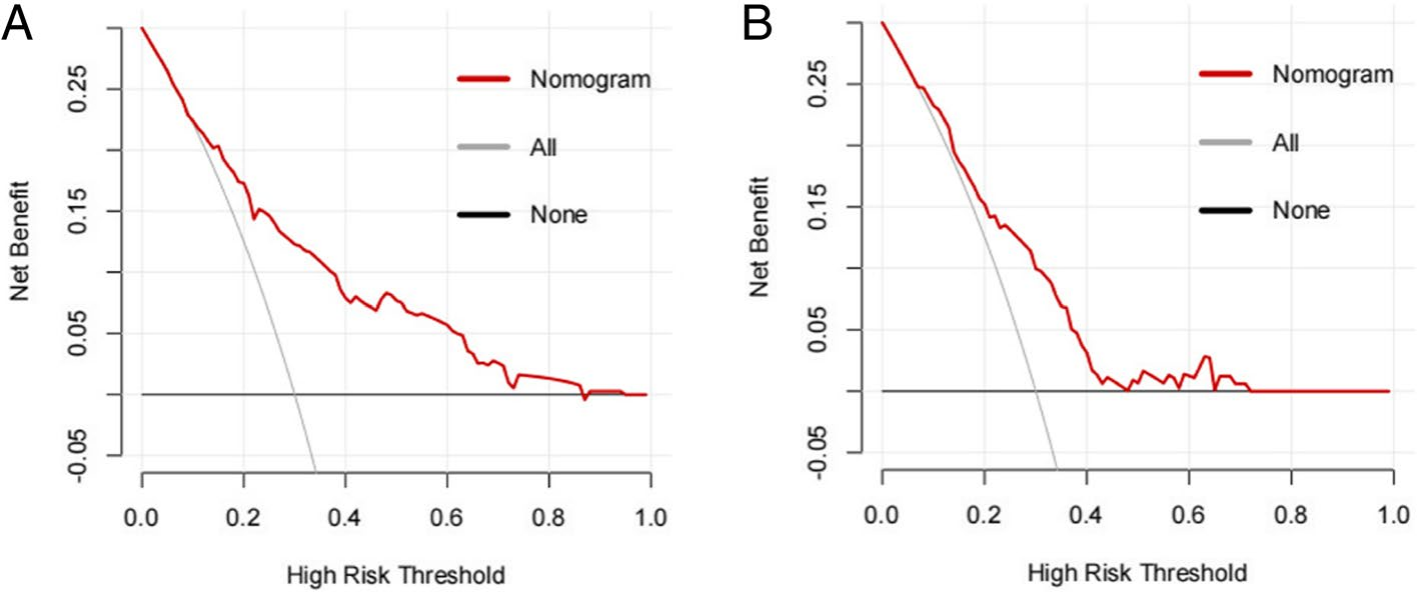
Decision curve analysis for CLNM in PTMC patients in validation cohort. The y-axis represents the net benefit. The red line represents the nomogram of CLNM. The grey line displays the assumption that all patients have CLNM. The black line represents the assumption that no patients have CLNM. PTMC:papillary thyroid microcarcinoma;CLNM:central lymph node metastasis

Furthermore, the AUC was 0.774 (95% CI: 0.723 to 0.826) for training cohort (Fig. 3a) while 0.709 (95% CI: 0.626 to 0.793) for validation cohort (Fig. 3b). As shown in the Hosmer-Lemeshow’s test results, the *p*-values were more than 0.05 in training cohort and validation cohort, and the Chi-square value were 5.0464 and 6.0584 in the training and validation cohorts. The calibration curve of CLNM nomogram suggested strong agreement between the training cohort (Fig. 4a) and the validation cohort (Fig. 4b), which indicated the high discriminating ability and consistency. Based on DCA, this model was better than those models where either all patients were treated or none underwent treatment (Fig. 5a, b).

### Risk stratification according to the risk scores and comparison of positive rate of CLNM

In the training cohort, there were 121 patients in the low-score-risk group, 174 in the medium-score-risk group, and 87 in the high-score-risk group (Table 4). In the mutual comparison of CLNM positive rates with different score-risk stratification, the *P* values were less than 0.05, which suggested that the model can distinguish patients according to the risk level (Table 5).

**Table 4:** Risk stratification in the training cohort

		count	*M*	*SD*	Min	Max	Q2	Q1	Q3
Training cohort	Low-score-risk	121	105.47	35.01	0	151.2	112.04	76.03	127.9
	Medium-score- risk	174	203.98	31.58	162.42	259.47	188.08	175.16	242.22
	High-score- risk	87	318.81	47.58	266.19	478.97	322.59	274.46	342.22

**Table 5:** comparison of positive rates in CLNM with different risk stratification in the training cohort

	Positive rate of CLNM	*X*^2^	*P* value	*SMD*
Low-score-risk	11 (9.09%)a	68.277	< 0.001	1.003
Medium-score- risk	49 (28.16%)b			
High-score- risk	54 (62.07%)c			

*Note*: There is no significantly statistical difference between the two groups with the same letter

In the validation cohort, 53 patients in the low-score-risk group, 72 in the medium-score-risk group and 39 in the high-score-risk group (Table 6). In the comparison between low-score-risk and middle-score-risk groups, the difference was statistically significant. But in the comparison between middle risk and high risk group, the P value was greater than 0.05, no significant differences were observed for the positive rate of CLNM between the training and validation cohorts. (Table 7)

**Table 6:** Risk stratification in the validation cohort

		count	*M*	*SD*	Min	Max	Q2	Q1	Q3
Validation cohort	Low-score-risk	53	106.9	32.13	36.88	146.56	112.04	76.03	140.51
	Medium-score- risk	72	199.12	32.37	151.2	251.2	188.08	175.16	237.59
	High-score- risk	39	315.82	43	266.19	413.62	316.55	274.46	342.22

**Table 7:** Comparison of positive rates in CLNM with different risk stratification in the validation cohort

	Positive rate of CLNM	*X*^2^	*P* value	*SMD*
Low-score-risk	8 (15.09%)a	10.607	0.005	0.586
Medium-score- risk	23 (31.64%)b			
High-score- risk	18 (46.15%)b			

*Note*: There is no significantly statistical difference between the two groups with the same letter

## Discussion

PTC shows an increasing incidence over the past 10 years, predominantly because of the increased detection of PTMC. Even though PTMC is the indolent cancer, some studies discover that PTMC cases with concurrent CLNM have dismal prognostic outcome and disease relapse.

Currently, one of the dilemmas in the management of PTMC patients is to determine whether pCLND should be performed. As found in the meta-analysis including 25 studies, prophylactic neck dissection combined with thyroidectomy is the possibly safe and efficient procedure for treating PTC cases, as it significantly reduces local recurrence [21]. Su et al. [22] also showed similar results. Moreover, a study reported that pCND significantly reduced the recurrence rate but was accompanied by numerous adverse effects [23]. Giordano et al. [24] found that pCND increased the rates of transient and permanent hypoparathyroidism. Selected CND is not obviously advantageous in local recurrence and long-term survival but it helps to accurately predict the pathological stage of central neck lymph nodes, although it will lead to an increased incidence of permanent hypoparathyroidism [25].

PTMC usually has a relatively high frequency of CLNM; the incidence of CLNM ranged from 13 to 64% in previous studies [26–30], which was largely due to the different choice of surgical modalities. The prevalence of CLNM in our study was 29.8% (163/546), which was similar to the prevalence of CLNM in previous studies [31–33].

In this study, age (< 45 years), sex, tumor size, multifocality, capsular invasion, and HT to be the risk factors that independently predicted CLNM among PTMC cases, which was similar to the risk factors determined in previous studies [8, 34–36]. However, the significant CLNM predictive risk factors among PTMC were dissimilar partially.

In this study, age was an important predictive factor, especially in patients below 45 years. Previous studies have determined age (< 45 years) as an independent risk factor [34, 36], and we found similar results in this study. Jiwang et al. [8] found that CLNM within PTMC patients was higher among males and younger individuals. Additionally, a study showed that male with a younger age were associated with the higher risk of large LNM in clinically node-negative PTMC [37]. Ding et al. [38] also found that PTC in men is a more aggressive disease and may have a worse prognosis. Sex was statistically significant in the univariate analysis and the multivariable analysis. Sex, as a significant predictor of the risk of CLNM, was consistent between our study and most previous studies. The differences could be explained by ethnic, geographic, patient characteristics, and environmental factors. Therefore, multicenter and large-scale studies are needed to address the problem.

There was no definitive cutoff value for tumor size. Most studies have considered 0.5 cm to be the threshold of size, while Lee and colleagues found that tumors that are greater than 0.7 cm could be important in determining PTMC invasiveness [39]. Gong et al. [40] showed that larger tumors (> 0.85 cm) are more aggressive. The results in this study showed that the high occurrence of CLNM was associated with PTMCs greater than 0.65 cm. Thus, we assumed that larger tumors are probably more aggressive.

In our study, the capsular invasion was shown to be an independent risk factor for predicting CLNM. Previous reports have indicated that extrathyroidal extension (ETE) is an independent predictor of CLNM [34, 35]. But the assessment of extrathyroidal extension remains problemic due to the anatomical features of the thyroid gland, there have no definitive standard about capsular invasion and extrathyroidal extension [34, 35]. Thus, our study take capsular invasion diagnosed by preoperative US as the predictor. The rate of capsular invasion of PTMC in our study was 18.4%, but it varied considerably among studies [34, 35]. The distinction in the incidence could be accounted for by different diagnostic criteria. Additionally, Ruiz Pardo et al. [41, 42] reported that the ETE of PTMC is a factor that indicates worse prognosis and is associated with the presence of metastatic lymph nodes and lower disease-free survival.

As shown in our study, multifocality was also found to be a important predictor of CLNM. Some authors reported multifocality as the the risk factor to independently predict CLNM among cN0 PTMC cases [8, 35, 43]. Additionally, Xue et al. [36] proposed that multiple cancer foci were independent risk factor for lateral lymph node metastasis (LLNM) in patients with PTMC.

PTC complicated by HT has been discovered in the past 20 years, and the association between the two diseases has been a topic of discussion [44].PTC cases have a remarkably increased HT rate compared with patients who have nonmalignant thyroid tumors [45]. In this study, HT was related to CLNM. HT rate was 18.50% among PTMC patients, and the rate of CLNM was 12.30% in the HT group, which was lower compared to that in the non-HT group (87.70%). Kim et al. [45] reported that HT, related to PTC, might protect against CLNM. Liang et al. [44] also found that PTC cases who had HT had low rates of central and lateral LNM (19.6% vs. 9.5%). The autoimmune response to thyroid-specific antigens in patients with HT might be involved in the destruction of cancer cells that express thyroid-specific antigens in PTC, thus preventing recurrence and LNM. Nonetheless, Jeong et al. [46] found that central LNM was not different in PTC cases with HT compared with those with no HT. The correlation between the prognostic outcomes of the two diseases in PTMC is still unclear.

Few studies have predicted CLNM among PTMC cases based on the nomogram. In the present work, a nomogram was established to predict CLNM incidence among PTMC cases based on six independent influencing factors. As reported, an AUC value over 0.7 implies good discriminating ability. In our study, the AUC for the training and validation cohorts were 0.774 and 0.709, respectively. The calibration plots showed a good agreement between the actual probability and the predicted probability of CLNM in all cohorts. The decision curve analysis also demonstrated the potential application value of our nomogram. As suggested by DCA, the as-constructed nomogram might be applied in clinic. These results suggested that the as-constructed nomogram had a high discriminating capacity for CLNM detection among PTMC cases. Furthermore, we achieved a remarkable achievement compared to other similar models [47–49]. According to our nomogram, we calculated the risk scores of each patient, and patients were also stratified into different subgroup by their risk scores.

Our study had several limitations. First, this was a retrospective study, and the data used to construct the model came from a single center. Second, although 382 and 164 cases were enrolled in training and validation cohorts, respectively, further research is warranted, in particular those at other institutions, so as to assess whether our results are applicable in the external population. Third, we collected data only on representative ultrasonic features; the future prediction model should include more features from preoperative genotypes and representative images from FNAB cases. Despite these disadvantages, our nomogram showed favorable discriminating capacity as well as internal verification in predicting CLNM.

Thus, before making treatment decisions, we recommend a strict preoperative evaluation, especially for patients with a high nomogram score. The cutoff point can depend on how the patients and doctors evaluate and perceive risk. We conclude that our nomogram is a practical and objective tool for providing evidence for both physicians and patients.

## Conclusion

In our study, we presented a new nomogram, which might be used to identify CLNM incidence among PTMC cases, and thus, help physicians and patients to make an informed choice regarding surgery. For patients with a high score on the nomogram, clinicians may consider pCND and rigorous evaluation of the patient after the operation.

## Data Availability

All data generated or analysed during this study are included in this published article.

## Abbreviations

PTC: Papillary thyroid cancer
TC: Thyroid cancer
TSH: Thyroid stimulating hormone
US: Ultrasonography
FNAB: Fine-needle aspiration biopsy
PTMC: Papillary thyroid microcarcinoma
CLNM: Central lymph node metastasis
PCND: Prophylactic central neck dissection
HT: Hashimoto’s thyroiditis
AJCC: American Joint Committee on Cancer
SD: Standard deviation
OR: Odds ratio
CI: Confidence intervals
AUC: The receiver operating characteristic curve
