# Correction: A novel tool for predicting the risk of central lymph node metastasis in patients with papillary thyroid microcarcinoma: a retrospective cohort study

**DOI:** 10.1186/s12885-022-09768-x

**Published:** 2022-06-24

**Authors:** Qian-wen Luo, Shan Gao, Xiao Lv, Si-jia Li, Bo-fang Wang, Qing-qing Han, Yun-peng Wang, Quan-lin Guan, Tao Gong

**Affiliations:** 1 The First Clinical Academy of Lanzhou University, Lanzhou, 730000 Gansu China; 2 Department of Gland Surgery, HeBei General Hospital, Shijiazhuang, 050051 HeBei China; 3 Department of Cardiovascular Medicine, People’s Hospital of Dongxihu District, Wuhan, 430040 Hubei China; 4 The Second Clinical Academy of Lanzhou University, Lanzhou, 730000 Gansu China; 5 Department of surgical oncology, The Lanzhou University First Hospital, Lanzhou, 730030 Gansu China

**Correction: BMC Cancer 22, 1-12 (2022)**

**https://doi.org/10.1186/s12885-022-09655-5**

Following publication of the original article [1], the authors identified an error in the affiliations.

Professor Quan-lin Guan is affiliated to Lanzhou University First Hospital, and Professor Tao Gong is affiliated to HeBei General Hospital. The following is the correct format:

Qian-wen Luo1; Shan Gao2; Xiao Lv3; Si-jia Li2; Bo-fang Wang4; Qing-qing Han2; Yun-peng Wang4; Quan-lin Guan 5*; Tao Gong2*

1 The First Clinical Academy of Lanzhou University, Lanzhou , Gansu 730000, China

2 Department of Gland Surgery, HeBei General Hospital, Shijiazhuang, HeBei 050051, China

3 Department of Cardiovascular Medicine, People's Hospital of Dongxihu District, Wuhan, Hubei 430040, China

4 The Second Clinical Academy of Lanzhou University, Lanzhou 730000, Gansu, China

5 Department of surgical oncology, The Lanzhou University First Hospital, Lanzhou, Gansu 730030, China
